# The singular epidemiology of HPV infection among French Guianese women with normal cytology

**DOI:** 10.1186/s12889-017-4181-3

**Published:** 2017-03-24

**Authors:** Antoine Adenis, Valentin Dufit, Maylis Douine, Fatiha Najioullah, Vincent Molinie, Dominique Catherine, Odile Kilié, Nadia Thomas, Jean Luc Deshayes, Paul Brousse, Hatem Ben Amor, Remy Pignoux, Gabriel Carles, Claire Grenier, Vincent Lacoste, Raymond Cesaire, Mathieu Nacher

**Affiliations:** 1 Centre d’Investigation Clinique Antilles-Guyane, CIC INSERM 1424, Centre hospitalier de Cayenne, Rue des flamboyants, 97300 Cayenne, French Guiana; 2 Laboratoire de Virologie, CHU de la Martinique, Fort de France, Martinique; 3 Laboratoire d’anatomopathologie, CHU de la Martinique, Fort de France, Martinique; 4 Service de Gynecologie Obstétrique, Centre hospitalier de Cayenne, Cayenne, French Guiana; 5 AGDOC Association de Dépistage Organisé des Cancers de Guyane, Cayenne, French Guiana; 6 Département des Centres délocalisés de prévention et de soins, Centre Hospitalier de Cayenne, 97300 Cayenne, French Guiana; 7 Service de Gynecologie Obstétrique, Centre Hospitalier de l’Ouest Guyanais, Saint Laurent du Maroni, Cayenne, French Guiana; 8 Laboratoire des interactions virus Hôtes, Institut Pasteur de la Guyane, Cayenne, French Guiana

**Keywords:** Human papillomavirus, Genotype, Normal cytology, Prevalence, French Guiana

## Abstract

**Background:**

In French Guiana, cervical cancer is the second most frequent cancer in females. The objective of the present study was to describe the prevalence of HPV infections in women with normal cervical cytology living in the remote villages of French Guiana.

**Methods:**

Before the study, the study team communicated in the remote villages on the importance of screening. All women from the target population were offered to participate. They signed informed consent during inclusion and then had a concomitant HPV-test and cervical smear. Only women with normal cytology and a good quality smear were analyzed. The detection of HPV-DNA was performed using the GREINER-BIO-ONE kit.

**Results:**

Overall, 27.2% of women with normal cervical cytology had a positive HPV-test. There was a U-shaped evolution of prevalence with women over 50 years having the highest HPV prevalence, followed by the 20 to 29 years group. The most prevalent HPV genotypes were HPV 53(3.52%), 68(3.33%), 52(2.59%), 31(2.22%) and 16 (1.85%). The proportion of HPV 16 among HPV-infected women was 6.8%.

**Conclusions:**

HPV prevalence in cytologically normal women was very high. The most prevalent genotypes were very different from what is usually described in the world, and notably in South America.

## Background

Cervical cancer is caused by the Human Papilloma Virus. In French Guiana, the standardized incidence rate of cervical cancer is 30.3 per 100,000 women. It is the second most frequent cancer in females [1] and causes significant mortality [2]. These figures show that the epidemiology of cervical cancer in French Guiana, a French territory, is South American rather than French [3].

French Guiana is covered by primary forest and has a population of 240,000 inhabitants, mostly living along the coastline. However, part of the population lives in the interior of French Guiana and has reduced access to care when compared to persons living in the main cities. Studies have shown that persons start their sexual life early and have a greater number of sex partners than reported in mainland France. This presumably favors the sexual transmission of this virus [4, 5].

Vaccination against HPV is now recommended in France, and thus in French Guiana (bivalent or tetravalent vaccines have been commercialized since 2007) [6]. The opportunistic screening of women aged 25–65 years is recommended using cytological examination of cervico-vaginal smears. In France, the HPV test is only reimbursed when cytological examination reveals ASCUS lesions [7].

In French Guiana, efforts to conduct organized screening have been implemented in coastal areas, but not in the interior where isolation and lack of staff make it more difficult. Throughout the world, a number of studies have described the prevalence and risk factors of HPV in cytologically normal women [8–12]. Several studies in this particular subgroup have been pooled and have allowed to publish a meta analysis summarizing the findings and the marked regional differences regarding prevalence and the most prevalent genotypes [13, 14]. Chronic cervical inflammation may impair local immunity and influence the relative penetrance of other HPV genotypes than HPV 16 [13]. This target population may thus be more suitable for global comparisons.

In French Guiana, weconducted a study between 2012 and 2014 to determine the prevalence of HPV infection in women aged 20–65 living in the remote villages along the border rivers between French Guiana and Suriname or French Guiana and Brazil. Women had both a cervical smear and an HPV test. The objective of the present report was thus to describe the prevalence and risk factors of HPV infections in women with normal cervical cytology, and to compare these results with other regions of the world.

## Methods

### Study population

The cross sectional study took place between December 2012 and September 2014. The study population consisted of women aged 20–65 years having previously had sexual activity living in the remote villages on the Maroni and Oyapock rivers. Among these women only those with normal cervical cytology (Bethesda 2001 criteria) were included in the analysis.

Women with a history hysterectomy, pregnant women (>3 months pregnant) were not included. Women with abnormal cervical cytology were not included. Smears of insufficient quality (with an absence of endocervical cells) were excluded.

The detection of HPV DNA was performed using the GREINER-BIO-ONE kit at the Virology laboratory in Fort de France University Hospital. This kit allows the identification of high risk HPV genotypes 16, 18, 31, 33, 35, 39, 45, 51, 52, 53, 56, 58, 59, 66, 68, 70, 73, 82 and low risk genotypes 6, 11, 40, 42, 43, 44, 55.

### Data collection

A short questionnaire was used to collect socio economic and demographic data, and the gynecological and obstetrical history. Vaccines against HPV being theoretically available since 2006 (tetravalent) or 2007(bivalent), all women were asked if they had been vaccinated against HPV.

### Study conduct

A study coordinator first went to the study sites to meet the key opinion leaders, local authorities (administrative and traditional). Communication was performed in all villages in order to sensitize the local populations about the public health problem of cervical cancer before beginning the inclusion of study patients. Health center workers were also informed and the logistical conditions of the health centers were assessed to ensure that the study was feasible. Three weeks before the research started, radio messages were aired several times a day in local languages informing the population about when the study would take place in the village. A study coordinator first went to the study sites to meet the key opinion leaders, local authorities. Three weeks before the research started, radio messages were airedseveral times a day in local languages informing the population about when the study would take place in the village. When the study team arrived, persons wishing to do an HPV test and cervical cytology came to the health center. The project was explained to them, the questionnaire was then filled and samples were taken using the ThinPrep® kits. Samples were stored in a cooler until the end of the mission. The samples were then sent to Fort de France Hospital’s Virology laboratory, in Martinique, where an automated method was used for DNA extraction and genotyping. Sample DNA extraction was performed using a minimum of 2 ml in a liquid phase, followed by PCR for amplification, and genotyping using a kit that could discriminate between 20 genotypes, (HR and potentially oncogenic genotypes) and identify multiple infections.

When the HPV test was positive and when cytology was negative, a gynecological follow up was recommended to verify if HPV positivity had disappeared or if cytological lesions had appeared.

### Ethical and regulatory aspects

For study participants, the HPV test and cytology were free of charge. All included subjects gave written informed consent to participate in the study and for the publication of its results. Regulatory and ethical approval were given by the Comité d’Evaluation Ethique de l’Inserm (CEEI), approval n° 12–064; the Comité Consultatif sur le Traitement de l’Information en matière de Recherche dans le domaine de la Santé (CCTIRS), n° 12.310; and the Commission Nationale de l’Informatique et des Libertés (CNIL), n° 912,459.

### Data analysis

Prevalence was obtained by dividing the number of women with normal cytology infected by at least one HPV genotype (high grade or low grade) by the total number of women with normal cervical cytology. For specific genotypes, prevalence was obtained by the number of women infected with normal cytology by that specific genotype by the total number of women with normal cervical cytology. The direct standardization method was used to obtain the standardized HPV prevalence (using the world population as a reference population). The sample size required to detect a 30% prevalence with a 5% margin of error and a 95% confidence interval and an estimated population of 10,000 would be 313 persons, a figure which was exceeded.

Risk factors associated with HPV prevalence were determined using Generalized Linear Models using the log link and Poisson family. The Pearson goodness of fit test was used. The data was analyzed using STATA 13 software (College Station, Texas, USA).

## Results

Overall, a total of 643 women were included, of which 540 women had normal cytology. Sexual relations generally started at a young age, with 19.1% percent of the surveyed women having had their first sexual relation before the age of 15 years.

A large proportion (*N* = 147, 27.2%) of women with normal cytology had a positive HPV test. Table one shows a high HPV prevalence notably for the high risk viruses. The overall age-standardized prevalence was 28.9%**.** Table 1 shows there was a U shaped evolution of overall HPV prevalence by age and of high-risk-HPV prevalence by age, with women over 50 years of age at highest risk for HPV (40.8%), followed by the 20 to 29 years group (27.5%). Speaking Maroon languages was associated with a greater HPV prevalence, whereas speaking Portuguese was associated with a lower HPV prevalence. Other risk factors such as the number of sex partners, age at first sex, contraception use, parity, education, or smoking were not significantly associated with differences in HPV prevalence. None of the women declared receiving the HPV vaccine.

**Table 1:** HPV prevalence in women with normal cervical cytology living in the remote areas of French Guiana

			Proportion of women positive for HPV		
	N	Overall %	% any HPV	% HR	% LR
Total	540	100.0	27.2	17.9	8.7
Age (years)					
20-29	142	26.3	27.5	21.8	7.0
30-39	197	36.5	25.9	18.8	9.1
40-49	125	23.1	20.8	16.0	7.2
50-64	76	14.1	40.8	35.5	13.2
Education					
Never	183	33.9	32.8	27.3	10.9
Low	173	32.0	26.0	19.1	9.2
Intermediate and high	181	33.5	21.5	16.6	5.5
Missing	3	0.6	100.0	66.7	33.3
Native language					
Maroon languages	222	41.1	31.1	24.8	9.5
Amerindian languages	177	32.8	26.6	20.9	9.0
Portugueuse	98	18.1	20.4	16.3	6.1
Others	43	8.0	25.6	16.3	9.3
Age at first sexual intercourse					
< 15	103	19.1	25.2	20.4	5.8
15-17	191	35.4	24.1	18.8	6.8
>= 18	73	13.5	27.4	21.9	12.3
Missing	173	32.0	31.8	24.3	11.0
Number of sexual partners in previous year					
0	38	7.0	36.8	34.2	15.8
1	321	59.4	25.5	19.6	7.5
>=2	61	11.3	26.2	23.0	6.6
Missing	120	22.2	29.2	20.8	10.8
Parity					
0-1	89	16.5	29.2	27.0	4.5
2-3	162	30.0	22.8	16.0	9.9
4-5	131	24.3	26.7	22.1	6.1
> 5	158	29.3	31.0	22.8	12.0
Contraceptives					
Never	362	67.0	26.5	21.8	8.0
Oral	86	15.9	32.6	24.4	9.3
Others	92	17.0	25.0	16.3	10.9
Smoking					
Yes	29	5.4	27.6	13.8	13.8
No	511	94.6	27.2	21.7	8.4

Table 2 shows that HPV 53 and 68 were the 2 most frequent high risk viruses. HPV16 was the 5th most frequent genotype in women of all ages with normal cervical cytology. However, it was the most frequent in women aged 30–39 years with normal cervical cytology. It seemed less frequent in women aged over 40 years. Overall, the prevalence of HPV 16 or 18 exceeded 3% (95% CI 2.1%–5.4%) of tested women with normal cervical cytology. Among women with normal cytology with any (low and high risk) HPV infection, the proportion of women infected with HPV 16 was 6.8% (95% CI 3.3%–12.1%). Among women with normal cytology with high risk HPV infection, the proportion of women infected with HPV 16 was 8.7% (95% CI 4.2%–15.4%).

**Table 2:** Prevalence of different HPV genotypes in women with normal cervical cytology living in remote areas of French Guiana

	Age < 30 years (*n* = 142)	Age 30-39 years (*n* = 197)	Age >= 40 years (*n* = 201)	All ages (*n* = 540)	Prevalence
	No	No	No	No	%
HPV HR type					
53	6	5	8	19	3.52
68	3	4	11	18	3.33
52	5	2	7	14	2.59
31	4	3	5	12	2.22
16	3	6	1	10	1.85
56	5	2	3	10	1.85
18	1	3	5	9	1.67
51	1	5	3	9	1.67
58	4	2	1	7	1.30
39	2	2	3	7	1.30
70	0	3	4	7	1.30
45	0	3	2	5	0.93
66	2	2	0	4	0.74
35	0	1	2	3	0.56
73	2	0	1	3	0.56
82	1	1	0	2	0.37
33	0	0	1	1	0.19
59	0	0	1	1	0.19
HPV LR type					
44 / 55	3	9	12	24	4.44
42	3	4	3	10	1.85
6	3	4	0	7	1.30
40	1	1	3	5	0.93
43	0	0	2	2	0.37
11	0	0	1	1	0.19

Overall, 115 women (21.3%) had single HPV infections, 23 (4.3%) had double infections, and 9 (1.7%) had 3 or more different HPV genotypes. Having more than one HPV genotype was more frequent in women over 50 years of age (11/20) relative to those <= 50 (21/95) (crude OR = 2.5 (95% CI 1–6.4), *p* = 0.037.

Table 3 shows multiple regression analysis models predicting HPV positivity in women with normal cervical cytology. No variables significantly predicted HPV positivity.

**Table 3:** Generalized linear model analyzing potential risk factors for HPV infection in women with normal cervical cytology living in the remote areas of French Guiana

	Positive for HPV detection		Positive for HPV HR type		Positive for HPV LR type	
	No	Prevalence ratio^a^	No	Prevalence ratio^a^	No	Prevalence ratio^a^
Age (years)						
20-29	39	1.6 (0.7–3.4)	31	1.6 (0.7–3.7)	10	1.4 (0.3–5.3)
30-39	51	1.3 (0.6–2.5)	37	1.4 (0.6–3.1)	18	1.1 (0.3–3.2)
40-49	26	1 (reference)	20	1 (reference)	9	1 (reference)
50-64	31	1.9 (0.9–4.5)	27	2 (0.8–5.2)	10	1.6 (0.4–7.2)
Education						
Never	60	1 (reference)	50	1 (reference)	20	1 (reference)
Low	45	1 (0.5–2.3)	33	1 (0.5–2)	16	0.8 (0.3–2.6)
Intermediate and high	39	0.7 (0.4–1.5)	30	0.7 (0.3–1.6)	10	0.4 (0.1–1.6)
Native language						
Maroon languages	69	1 (0.5–2.1)	55	0.9 (0.4–2)	21	0.9 (0.2–3.7)
Amerindian languages	47	1.1 (0.5–2.3)	37	1.1 (0.5–2.4)	16	1.3 (0.3–4.6)
Portugueuse	20	1 (reference)	16	1 (reference)	6	1 (reference)
Others	11	1.2 (0.5–2.8)	7	0.7 (0.3–1.6)	4	2.4 (0.6–9.7)
Age at first sexual intercourse						
< 15	26	1 (reference)	21	1 (reference)	6	1 (reference)
15-17	46	1.0 (0.6–1.8)	36	0.9 (0.5–1.6)	13	1.3 (0.5–3.5)
>= 18	20	1.2 (0.6–2.6)	16	1.1 (0.5–2.1)	9	2.8 (0.9–9)
Number of sexual partners in previous year						
0	14	1 (reference)	13	1 (reference)	6	1 (reference)
1	82	0.8 (0.4–1.9)	63	0.6 (0.3–1.5)	24	0.8 (0.2–3.3)
>=2	16	1.1 (0.4–2.7)	14	0.9 (0.3–2.5)	4	1.3 (0.2–7)
Parity						
0-1	26	1 (reference)	24	1 (reference)	4	1 (reference)
2-3	37	0.9 (0.4–1.7)	26	0.8 (0.4–1.6)	16	1.4 (0.3–5.6)
4-5	35	1.2 (0.6–2.4)	29	1 (0.5–2.1)	8	2 (0.5–8.5)
> 5	49	1.3 (0.6–2.7)	36	0.9 (0.4–2)	19	2.6 (0.5–12.4)
Contraceptives						
Never	96	1 (reference)	79	1 (reference)	29	1 (reference)
Oral	28	1.1 (0.6–2.1)	21	1.1 (0.5–2.2)	8	1 (0.3–3.1)
Others	23	0.8 (0.4–1.7)	15	0.6 ( 0.2–1.4)	10	1.6 (0.5–4.5)
Smoking						
No	139	1 (reference)	111	1 (reference)	43	1 (reference)
Yes	8	0.7 (0.3–1.9)	4	2.1 (0.5–9.3)	4	0.2 (0.1 - 0.9)

^**a**^GLM using the log link and Poisson family. Goodness of fit Pearson test >0.9 for all models

## Discussion

The apparent variation in the relative importance of different HPV genotypes requires further research including studies from regions where data is lacking, and reanalyzing existing data to better understand the observed patterns. [13] The published studies included in the pooled analysis and in the metaanalysis used the international cancer research association standard methodology for HPV prevalence surveys. Our sampling design was tailored to fit the logistical constraints of the remote villages in French Guiana. This may have introduced some differences in the prevalence estimations, but it seems unlikely that the relative frequency of different genotypes would be affected. [13].

Overall, the observed crude and standardized (28.9%) HPV prevalence among cytologically normal women in this French territory was among the highest reported in the world. It was notably much higher than in other areas of South America (standardized prevalence = 14.3%). However, the published reviews in the group of cytologically normal women in South America [13, 14] did not involve women living in the Amazon basin which is ethnically and socioeconomically different from Argentina (standardized prevalence = 16.3%) [15], Chile (standardized prevalence = 11.9%) [16], or Colombia (standardized prevalence = 13.9%) [17]. Indeed a recent study in the Brazilian State of Amazonas showed a very high HPV prevalence (crude prevalence in women with and without cytological anomalies = 39.7%) [18]. It is also worth noting that the mean age in women from Amazonas was 1.6 years younger than women from French Guiana. When looking at the most frequent genotypes, the results from rural French Guiana were also very different from pooled results from different areas of the world. HPV 16 was only found in 6.8% of HPV-positive women with normal cervical cytology, whereas it has been reported to be around 21.4% in South America [13], 13.7% in Mexico [19], and 9.8% in Costa Rica [20], and 25.5% in Europe, 24.1% in Canada [21], and 31.2% in the USA [13, 22]. Thus, the prevalence of HPV 16 in French Guiana was lower than but close to the prevalence in Mexico and Costa Rica. In French Guiana, HPV 16 was overall the 5th most frequent genotype whereas HPV 53 and HPV 68 were the 2 most frequent, which is a singularity when compared to other regions of the world where HPV 16 is the most frequent followed by HPV 58. HPV 31 was the 4th most frequent genotype in our study (2.2% of women with normal cytology) and, globally, it is the 3rd most common genotype (0.7% of all women with normal cytology). In rural Amazonas, where HPV prevalence was reported to be very high [18], the most frequent HPV genotypes were HPV 31, 68 and 53 in Macuxi and Wapishana women (which is similar as in our study), whereas for the Yanomani women, it was HPV 16, 31 and 18. However, this study included women with abnormal cytology and thus the comparison may not be appropriate [18].

These differences may reflect epidemiologic differences in transmission chains in these remote areas populated with ethnically homogenous populations with little admixture. A possible explanation for these differences in small isolated populations could be the founder effect, were a few individuals have spread these HPV genotypes. Immunologic differences between these ethnic groups (HLA allele frequencies in Amerindians versus Maroons for example [23]) could also influence the observed patterns of HPV genotypes. [24–27] Further studies should however test this hypothesis. As observed elsewhere [14], there was a U-shaped evolution of HPV prevalence with women over 50 years of age having the highest HPV prevalence, and also being more likely to be infected by more than one HPV genotype. However, given the early start of sexual life in this region, it is possible that HPV prevalence is also high in women under twenty years of age, who were not surveyed here.

The originality of this epidemiologic situation may result from the very singular situation of the isolated populations: different ancestral origins of the Maroon and Amerindian populations living in adjacent villages, with early sexual debut, rare sexual encounters between ethnic groups, different immunological susceptibilities, and different pools of HPV viruses. These results reemphasize [28] the potential interest of primary screening using HPV testing in this region where over a quarter of HPV positive women have normal smears and harbor high grade HPV genotypes. This however requires authorities to expand reimbursement to all targeted women and not only those with abnormal cytology, as is presently the case. The main HPV genotypes circulating also suggest that vaccination coverage, which is presently null, needs to be expanded. This implies operational aspects, and perhaps also suggests that the tetravalent vaccine should be replaced with the nonavalent HPV vaccine, which covers HPV 6, 11, 16, 18, 31, 33, 45, 52, and 58, because here HPV 52 was the third and HPV 31 the fourth most frequent high risk genotypes when HPV16 was fifth and HPV 18 seventh most common high risk genotypes.

## Conclusions

HPV prevalence in cytologically normal women was very high. The most prevalent genotypes were different from what is usually described in the world, and notably in South America beyond the Amazon basin. In a region where cervical cancer is frequent, vaccination against HPV and screening of women are important, but they must be adapted to the local epidemiology and made affordable for the populations.
